# Is androgen deprivation therapy associated with cerebral infarction in patients with prostate cancer? A Korean nationwide population‐based propensity score matching study

**DOI:** 10.1002/cam4.2325

**Published:** 2019-06-10

**Authors:** Bum Sik Tae, Byeong Jo Jeon, Hoon Choi, Jae Hyun Bae, Jae Young Park

**Affiliations:** ^1^ Department of Urology Korea University Ansan Hospital, Korea University College of Medicine Ansan Korea

**Keywords:** androgen deprivation therapy, cerebral infarction, nationwide population‐based study, prostate neoplasm

## Abstract

**Purpose:**

Previous studies have suggested that androgen deprivation therapy (ADT) is associated with cerebral infarction. However, conflicting results have been reported by other researchers. The aim of this study was to evaluate the association between ADT and cerebral infarction in patients with prostate cancer (PC) using big data.

**Materials and Methods:**

Using information from the National Health Insurance Service database representative of the entire Korean adult PC population (n = 206 735), data regarding ADT and cerebral infarction between 2009 and 2016 were analyzed. Adjusted hazard ratios for cerebral infarction associated with ADT were estimated using propensity score‐matched Cox proportional hazards models and Kaplan‐Meier survival analyses.

**Results:**

The final cohort comprised 36 146 individuals with PC, including 24 069 men (66.6%) who underwent ADT. During the mean follow‐up of 4.1 years, 2792 patients were newly diagnosed with cerebral infarction. In the unmatched cohort, there was a significant difference in the annual incidence of cerebral infarction between the ADT and non‐ADT groups (22.8 vs 14.6 per 1000 person‐years, respectively). However, there was no significant difference between the ADT and non‐ADT groups in the matched cohort (14.9 vs 14.6 per 1000 person‐years). The adjusted hazard ratio for cerebral infarction for PC patients who underwent ADT was 1.045 (95% CI 0.943‐1.159; *P* = 0.401) compared with those who did not undergo ADT. In addition, the cumulative duration of ADT was also not associated with an increased risk for cerebral infarction. However, older age, hypertension, diabetes, myocardial infarction, congestive heart failure, peripheral vascular disease, renal disease, dementia, and atrial fibrillation were revealed to be factors contributing to cerebral infarction.

**Conclusion:**

This nationwide population‐based study revealed that ADT was not associated with cerebral infarction after adjusting for potential confounders.

**Abbreviations**
ADT: Androgen deprivation therapy
CHF: Congestive heart failure
DM: diabetes mellitus
GnRH: gonadotropin‐releasing hormone
HR: hazard ratio
HTN: Hypertension
ICD: International Classification of Disease
MI: myocardial infarction
NHIS: National Health Insurance Service
PC: Prostate cancer
PSA: prostate‐specific antigen
PVD: peripheral vascular disease
SSRI: selective serotonin reuptake inhibitors

## 1 INTRODUCTION

Prostate cancer (PC) is one of the most common cancers among men worldwide, and its incidence is rising rapidly in Korea.^1^, ^2^ There are a variety of treatment options for localized PC, including radical prostatectomy and radiotherapy.^3^ However, androgen deprivation therapy (ADT) is the primary treatment method for advanced and metastatic PCs.^4^ ADT suppresses testosterone levels, delays the progression of PC, and improves survival.^5^

However, the suppression of testosterone levels has been associated with metabolic changes including dyslipidemia, insulin resistance, and modification of body composition.^6^, ^7^ Numerous recent studies have reported that ADT is associated with several adverse effects such as metabolic syndrome, cardiovascular diseases, dementia, and psychiatric disorders.^5^, ^8^, ^9^, ^10^, ^11^ Moreover, these conditions are known to be associated with a risk for cerebral infarction. A previous study reported that ADT is associated with the risk for cerebral and cardiovascular ischemic events.^12^ In addition, Azoulay et al, reported that the risk for stroke varied according to the type of ADT.^13^ In a study from Asia, Teoh et al, reported that ADT was correlated with cerebral ischemic strokes in a cohort of 452 patients.^14^ However, these previous studies had limitations in design in that they did not compensate for morbidity between the ADT and non‐ADT groups. Moreover, conflicting results have been reported by other researchers.^15^, ^16^ Therefore, this study aimed to evaluate the association between ADT and cerebral infarction in patients with PC using nationwide population‐based data.

## 2 MATERIALS AND METHODS

### 2.1 Ethics statement

The Institutional Review Board (IRB) of Korea University Ansan Hospital (Ansan, Korea) approved this study (IRB No. 2018AS0158), which was performed in accordance with the principles of the Declaration of Helsinki. The National Health Insurance Service (NHIS) database does not allow access to information identifying patients.

### 2.2 Study population selection

Approximately 97% of Koreans are enrolled in the mandatory universal health insurance program and receive comprehensive medical care. This analysis was conducted using the NHIS claims database, which includes actual claims data for the entire Korean population (approximately 50 million individuals).^17^ The *International Classification of Diseases, Tenth Revision*, Clinical Modification (ICD‐10‐CM) codes were used to identify diagnoses.

### 2.3 Definition of outcomes and covariates

**Figure 1:**
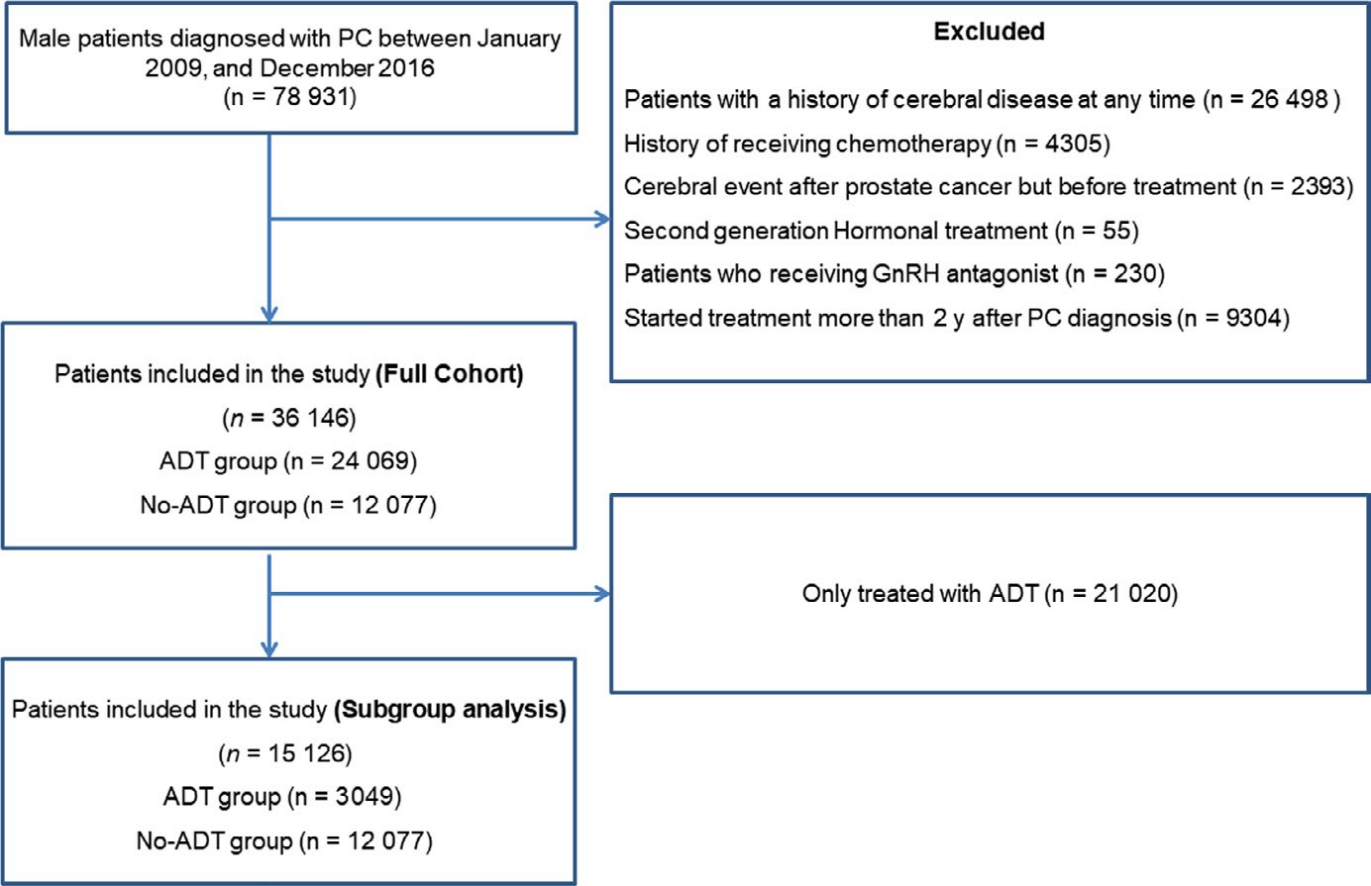
Study flow diagram of the cohort of patients newly diagnosed with prostate cancer in the Korean national health insurance system between 2009 and 2016. ADT, androgen deprivation therapy

Korean patients ≥40 years of age with an ICD‐10 diagnosis code for PC (C61) between 2009 and 2016 were included in this study. ADT included the administration of oral anti‐androgens (cyproterone acetate, flutamide, and bicalutamide), gonadotropin‐releasing hormone (GnRH) agonists (leuprolide, goserelin, and triptorelin), estrogens (estramustine), and undergoing bilateral orchiectomy (Supplemental Table S1). A time‐dependent exposure definition was used, which enabled patients to transition from a period of nonexposure to a period of ADT exposure. Patients without exposure to ADT were considered as the control group only if they attended follow‐up visits after the median time of undergoing ADT in the exposed group. Patients who were diagnosed with PC before 2009 were excluded. Patients with a history of cerebral disease (ischemic stroke, cerebral hemorrhage, or transient ischemic attack at any time before diagnosed PC), previous ADT treatment, but developed any type of cerebral disease before ADT, those treated with androgen receptor targeting agents, such as abiraterone acetate or enzalutamide, or GnRH antagonist, individuals with castration‐resistant PC, and those who received chemotherapy after their diagnosis were excluded from this study (Figure 1).

Cerebral infarction was categorized using the ICD‐10 diagnostic codes (Supplemental Table S2). To avoid selection bias and overestimation due to the inclusion of subjects with cerebral infarction, only patients with at least one diagnosis of cerebral infarction during hospitalization or two or more diagnoses in the outpatient clinic were included. Among patients undergoing ADT, incident cerebral infarction was ascertained after the start of ADT and at least six months after the diagnosis of PC. Among patients not undergoing ADT, incident cerebral infarction was ascertained six months after the diagnosis of PC.

Adjustment covariates included: age at PC diagnosis; chronic use of medication such as anticoagulant, antiplatelet, statin, selective serotonin reuptake inhibitor (SSRI), or antipsychotic medications; and a history of hypertension (HTN), any type of diabetes mellitus (DM), congestive heart failure (CHF), myocardial infarction (MI), peripheral vascular disease (PVD), malignant neoplasm, renal disease (RD), dementia, or atrial fibrillation (as defined in Supplemental Tables S1 and S2).

### 2.4 Subgroup analysis and statistical analysis

The National Comprehensive Cancer Network and the European Association of Urology guidelines recommend ADT as the first management option for PC patients with metastatic disease.^3^, ^4^, ^18^ To analyze only patients with localized PC at the time of diagnosis, those who underwent ADT only, without an operation or radiotherapy within six months of diagnosis, were excluded from subgroup analysis.

The start of the follow‐up period was defined as the time of PC diagnosis; the end of the follow‐up period was defined as the date of the last available record, either inpatient or outpatient, or the time of cerebral infarction diagnosis. Baseline characteristics were compared between the ADT and non‐ADT groups using the Student's *t* test or the *χ* ^2^ test. Hazard ratios (HRs) were calculated using multivariable‐adjusted Cox proportional hazards models to evaluate the effect of ADT on the risk for cerebral infarction. To adjust for comorbidity, 1:1 nearest‐neighbor propensity score matching was used without replacement. Variables included in the propensity score matching, and in the traditional multivariable‐adjusted Cox proportional hazards models, were as follows: age, use of medication (antiplatelet, anticoagulant, statin, SSRI, or antipsychotic medications), medical history (HTN, PVD, RD, DM, MI, CHF, dementia, atrial fibrillation), or history of malignant neoplasm. Kaplan‐Meier analysis was adopted to calculate the cumulative probability of remaining cerebral infarction‐free in the matched and unmatched cohorts. To assess the effect of ADT duration on cerebral infarction, Cox proportional hazards models were used to examine the risk for cerebral infarction among those with PC who underwent fewer than 12 months of ADT and ≥12 months of ADT compared with those who did not undergo ADT.^11^, ^19^, ^20^, ^21^ All statistical analyses were performed using SAS version 9.2 (SAS Institute, Cary, NC) for Windows (Microsoft Corporation, Redmond, WA).

## 3 RESULTS

### 3.1 Total cohort participated in the study

A total of 36 146 patients with PC, consisting of 12 077 in the non‐ADT group and 24 069 in the ADT group, met the inclusion criteria (Figure 1). The mean duration of follow‐up was 4.1 years and 2792 (7.72%) patients were newly diagnosed with cerebral infarction. There was a significant difference in the annual incidence of cerebral infarction between the ADT (22.8 per 1000 person‐years) and the non‐ADT (14.6 per 1000 person‐years) groups (*P* < 0.001) in the unmatched cohort. The ADT group was statistically significantly older, had more histories of MI, CHF, PVD, RD, and dementia, had more anticoagulant or antipsychotic use, and less statin use than the unmatched cohort (Table 1).

**Table 1:** Demographic characteristics of patients with prostate cancer, stratified by ADT (n = 36 146)

Variable	Full cohort			Propensity score–matched full cohort		
	No ADT (N = 12 077)	Received ADT (N = 24 069)	p value	No ADT (N = 11 252)	Received ADT (N = 11 252)	*P* value
*Age*	66.87 ± 6.62	73.45 ± 8.02	<0.001	67.25 ± 6.50	68.06 ± 7.13	0.786
<70 years	8355 (69.2%)	7382 (32.5%)	<0.001	6770 (59.3%)	6675 (59.3%)	0.999
≥70 years	3722 (30.8%)	16 237 (67.5%)		4482 (40.7%)	4487 (40.7%)	
*Medical history*						
Hypertension	6288 (52.1%)	12 305 (51.1%)	0.091	5787 (51.4%)	5771 (51.3%)	0.831
Diabetes	3050 (25.3%)	5995 (24.9%)	0.472	2797 (24.9%)	2871 (25.5%)	0.256
Prior cancer history	1676 (13.9%)	3264 (13.6%)	0.409	1586 (14.1%)	1575 (14.0%)	0.833
Myocardial infarction	189 (1.6%)	473 (2.0%)	0.007	165 (1.5%)	178 (1.6%)	0.479
Congestive heart failure	438 (3.6%)	1293 (5.4%)	<0.001	412 (3.7%)	414 (3.7%)	0.943
Peripheral vascular disease	966 (8.0%)	2135 (8.8%)	0.005	896 (8.0%)	905 (8.0%)	0.825
Renal disease	406 (3.7%)	930 (3.9%)	0.017	367 (3.3%)	372 (3.3%)	0.852
Dementia	224 (1.9%)	1272 (5.3%)	<0.001	224 (2.0%)	237 (2.1%)	0.541
Atrial fibrillation	385 (3.2%)	757 (3.2%)	0.827	337 (3.0%)	343 (3.1%)	0.815
*Medication status*						
Anticoagulants	116 (1.0%)	332 (1.4%)	0.001	116 (1.0%)	118 (1.0%)	0.825
Antiplatelets	2866 (23.9%)	5935 (24.7%)	0.112	2866 (25.5%)	2865 (25.5%)	0.999
Statins	2643 (21.9%)	4364 (18.1%)	<0.001	2643 (23.5%)	2643 (23.5%)	0.999
SSRI	73 (0.6%)	135 (0.6%)	0.606	73 (0.6%)	73 (0.6%)	0.999
Antipsychotics	70 (0.6%)	259 (1.1%)	<0.001	70 (0.6%)	70 (0.6%)	0.999
*Treatment*						
Radical prostatectomy	9830 (81.4%)	2310 (19.1%)	<0.001	9139 (81.2%)	1689 (15.0%)	<0.001
Radiotherapy	2782 (23.0%)	934 (7.7%)	<0.001	2618 (23.3%)	620 (5.5%)	<0.001
Follow‐up (day), mean (SD)	1567 ± 810	1353 ± 852	<0.001	1567 ± 810	1486 ± 872	0.004

Abbreviations: ADT: androgen deprivation therapy; SD: standard deviation.

However, there were no statistically significant differences among baseline characteristics between the ADT and non‐ADT groups in the propensity score‐matched cohort. In the matched cohort, there was no significant difference in the incidence of cerebral infarction between the ADT (14.9 per 1000 person‐years) and the non‐ADT (14.6 per 1000 person‐years) groups (*P* = 0.786). ADT was not associated with cerebral infarction (HR 1.045 [95% confidence interval (CI) 0.943‐1.159]; *P* = 0.401) (Table 2) in the multivariable analysis. However, old age (≥70 years, HR 1.735 [95% CI 1.562‐1.926]; *P* < 0.001) and a history of HTN (HR 1.313 [95% CI 1.171‐1.472]); *P* < 0.001), DM (HR 1.240 [95% CI 1.106‐1.391]; *P* = 0.002), MI (HR 1.558 [95% CI 1.114‐2.180]; *P* = 0.010), CHF (HR 1.481 [95% CI 1.190‐1.842]; *P* = 0.004), PVD (HR 1.259 [95% CI 1.069‐1.482]; *P* = 0.006), RD (HR 1.845 [95% CI 1.489‐2.287]; *P* < 0.001), dementia (HR 2.741 [95% CI 2.157‐3.483]; *P* < 0.001), and atrial fibrillation (HR 1.541 [95% CI 1.193‐1.990]; *P* = 0.001) were associated with the risk for cerebral infarction. However, a medication history of antiplatelet, statin, anticoagulants, SSRI, and/or antipsychotics use was not associated with a risk for cerebral infarction (*P* > 0.05).

**Table 2:** Multivariable cox regression for the association of covariates with cerebral infarction

Variable	Propensity score–matched full cohort	
	HR (95% CI)	*P* value
Age (≥70)	1.735 (1.562‐1.926)	<0.001
*Medical history*		
Hypertension	1.313 (1.171‐1.472)	<0.001
Diabetes	1.240 (1.106‐1.391)	0.002
Prior cancer history	1.018 (0.872‐1.190)	0.819
Myocardial infarction	1.558 (1.114‐2.180)	0.010
Congestive heart failure	1.481 (1.190‐1.842)	0.004
Peripheral vascular disease	1.259 (1.069‐1.482)	0.006
Renal disease	1.845 (1.489‐2.287)	<0.001
Dementia	2.741 (2.157‐3.483)	<0.001
Atrial fibrillation	1.541 (1.193‐1.990)	0.001
*Medication status*		
Anticoagulant	1.304 (0.882‐1.928)	0.184
Antiplatelet	1.086(0.957‐1.233)	0.200
Statin	1.023 (0.896‐1.168)	0.739
SSRI	0.959 (0.456‐2.017)	0.912
Antipsychotics	0.957 (0.491‐1.867)	0.898
*Treatment*		
Received ADT	1.045 (0.943‐1.159)	0.401
Radical Prostatectomy	0.862 (0.773‐0.961)	0.008
Radiotherapy	0.788 (0.656‐0.947)	0.011

Abbreviations: ADT: androgen deprivation therapy; HR: hazard ratio; CI: confidence interval.

**Figure 2:**
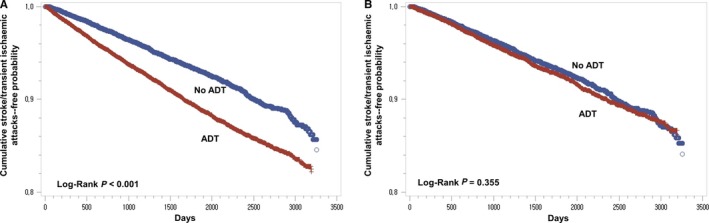
Kaplan‐Meier curves of cerebral infarction‐free probability in the total cohort (n = 36,146). (A) Kaplan‐Meier curves of cerebral infarction‐free probability in patients with prostate cancer who were exposed to androgen deprivation therapy (ADT, red) and who were not exposed to ADT (blue) in an unmatched cohort. (B) Kaplan‐Meier curves of cerebral infarction‐free probability in patients with prostate cancer who were exposed to ADT (red) and who were not exposed to ADT (blue) in a propensity score‐matched cohort

Kaplan‐Meier analyses revealed that the ADT group had a lower cumulative probability of remaining cerebral infarction‐free compared with the non‐ADT group in unmatched cohorts (*P* < 0.001 [log‐rank]) (Figure 2A). However, there was no significant difference in the cumulative probability of remaining cerebral infarction‐free among the ADT group versus the non‐ADT group in the propensity score‐matched cohort (*P* > 0.05 [log‐rank]) (Figure 2B).

### 3.2 Subgroup analysis

A total of 15 126 patients with PC, consisting of 12 077 in the non‐ADT group and 3049 in the ADT group, were included in the subgroup analysis (Figure 1). In the non‐matched cohort, a total of 946 (6.23%) patients were newly diagnosed with cerebral infarction. There was no significant difference in the annual incidence of cerebral infarction between the ADT (13.8 per 1000 person‐years) and the non‐ADT (14.6 per 1000 person‐years) groups (*P* = 0.874).

Patients undergoing ADT were statistically significantly older, had a history of cancer, and had less statin use in the unmatched cohort (Table 3). However, there were no statistically differences in baseline characteristics between the two groups in the propensity score‐matched cohort. In the matched cohort, no significant difference was observed in the annual incidence of cerebral infarction between the ADT (13.8 per 1000 person‐years) and non‐ADT (14.3 per 1,000 person‐years) groups (*P* = 0.672). ADT was not associated with cerebral infarction (HR 0.951 [95% CI 0.777‐1.164]; *P* = 0.625) in the multivariable analysis (Table 4).

**Table 3:** Demographic characteristics of patients with prostate cancer, stratified by ADT (n = 15 756)

Variable	Subgroup Cohort			Propensity Score–Matched Cohort		
	No ADT (N = 12 707)	Received ADT (N = 3049)	*P* value	No ADT (N = 3049)	Received ADT (N = 3049)	*P* value
*Age*	66.87 ± 6.62	68.10 ± 6.61	0.005	67.95 ± 6.45	68.10 ± 6.61	0.672
<70 years	8355 (69.2%)	1689 (55.0%)	<0.001	1695 (56.0%)	1689 (55.0%)	0.910
≥70 years	3722 (30.8%)	1360 (45.0%)		1354 (44.0%)	1360 (45.0%)	
*Medical history*						
Hypertension	6288 (52.1%)	1606 (52.7%)	0.549	1606 (52.7%)	1606 (52.7%)	0.999
Diabetes	3050 (25.3%)	794 (26.0%)	0.373	799 (26.2%)	794 (26.0%)	0.884
Prior cancer history	1676 (13.9%)	358 (11.7%)	0.001	358 (11.7%)	358 (11.7%)	0.999
Myocardial infarction	189 (1.6%)	45 (1.5%)	0.722	38 (1.2%)	45 (1.5%)	0.439
Congestive heart failure	438 (3.6%)	123 (4.0%)	0.288	116 (3.8%)	123 (4.0%)	0.644
Peripheral vascular disease	966 (8.0%)	242 (7.9%)	0.911	237 (7.8%)	242 (7.9%)	0.812
Renal disease	406 (3.4%)	90 (3.0%)	0.256	86 (2.8%)	90 (3.0%)	0.760
Dementia	224 (1.9%)	66 (2.2%)	0.265	69 (2.3%)	66 (2.2%)	0.794
Atrial fibrillation	385 (3.2%)	93 (3.1%)	0.698	86 (2.8%)	93 (3.1%)	0.595
*Medication status*						
Anticoagulants	116 (1.0%)	25 (0.8%)	0.471	25 (0.8%)	25 (0.8%)	0.999
Antiplatelets	2886 (23.9%)	716 (23.5%)	0.632	716 (23.5%)	716 (23.5%)	0.999
Statins	2643 (21.9%)	581 (19.1%)	0.001	581 (19.1%)	581 (19.1%)	0.999
SSRI	73 (0.6%)	11 (0.4%)	0.106	11 (0.4%)	11 (0.4%)	0.999
Antipsychotics	70 (0.6%)	24 (0.8%)	0.193	24 (0.8%)	24 (0.8%)	0.999
*Treatment*						
Radical prostatectomy	9830 (81.4%)	2310 (75.8%)	<0.001	2449 (80.3%)	2310 (75.8%)	<0.001
Radiotherapy	2782 (23.0%)	934 (30.6%)	<0.001	738 (24.2%)	934 (30.6%)	<0.001
Follow‐up (day), mean (SD)	1,567 ± 810	1,684 ± 833	<0.001	1,558 ± 807	1,684 ± 833	<0.001

Abbreviations: ADT: androgen deprivation therapy; SSRI: Selective serotonin reuptake inhibitors.

**Table 4:** Multivariable cox regression for the association of covariates with cerebral infarction in subgroup analysis

Variable	Propensity score–matched subgroup cohort	
	HR (95% CI)	*P* value
Age (≥70)	1.916 (1.564‐2.347)	<0.001
*Medical history*		
Hypertension	1.300 (1.040‐1.626)	0.021
Diabetes	1.216 (0.972‐1.520)	0.087
Prior cancer history	1.013 (0.735‐1.425)	0.891
Myocardial infarction	1.912 (1.230‐2.974)	0.004
Congestive heart failure	1.262 (0.815‐1.954)	0.296
Peripheral vascular disease	1.435 (1.046‐1.969)	0.025
Renal disease	2.234 (1.454‐3.433)	0.002
Dementia	2.536 (1.591‐4.043)	<0.001
Atrial fibrillation	1.512 (1.108‐2.054)	0.009
*Medication status*		
Anticoagulant	1.027 (0.309‐3.421)	0.965
Antiplatelet	1.065 (0.828‐1.371)	0.621
Statin	0.911 (0.699‐1.187)	0.490
SSRI	1.060 (0.439‐2.558)	0.897
Antipsychotics	0.567 (0.136‐2.355)	0.435
*Treatment*		
Received ADT	0.951 (0.777‐1.164)	0.625
Radical Prostatectomy	0.701 (0.385‐1.271)	0.242
Radiotherapy	0.629 (0.360‐1.098)	0.103

Abbreviations: ADT: androgen deprivation therapy; SSRI: Selective serotonin reuptake inhibitors; HR: hazard ratio; CI: confidence interval.

**Figure 3:**
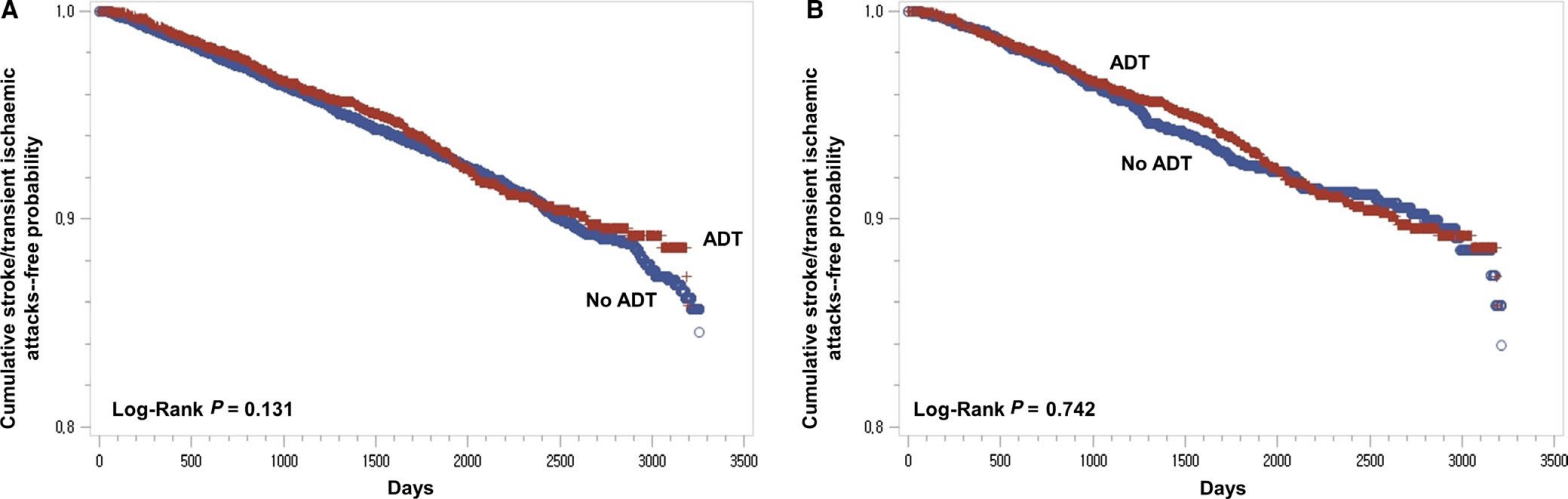
Kaplan‐Meier curves of cerebral infarction‐free probability in the subgroup analysis (n = 15 126). (A) Kaplan‐Meier curves of cerebral infarction‐free probability in patients with prostate cancer who were exposed to androgen deprivation therapy (ADT, red) and who were not exposed to ADT (blue) in an unmatched cohort. (B) Kaplan‐Meier curves of cerebral infarction‐free probability in patients with prostate cancer who were exposed to ADT (red) and who were not exposed to ADT (blue) in a propensity score‐matched cohort

In Kaplan‐Meier analyses, there was no significant difference in cumulative probability of remaining cerebral infarction‐free between the two groups in the unmatched cohort and in the propensity score‐matched cohort (*P* > 0.05 [log‐rank]) (Figure 3). Additionally, the duration of ADT was also not associated with cerebral infarction, both in propensity score matched full cohort and propensity score matched subgroup, excluding patients treated with ADT only without operation or radiotherapy (Table 5).

**Table 5:** Cox regression analysis for the association between ADT and cerebral infarction according to therapy duration

Duration of ADT use (Months)	Propensity score–matched, full cohort		Propensity score–matched, subgroup	
	HR (95% CI)	*P*‐value	HR (95% CI)	*P*‐value
No ADT	Ref	Ref	Ref	Ref
ADT < 12months	1.098 (0.993‐1.215)	0.069	0.920 (0.770‐1.098)	0.355
ADT ≥ 12 months	1.112 (0.969‐1.276)	0.131	0.966 (0.785‐1.189)	0.743

Abbreviations: ADT: androgen deprivation therapy; HR: hazard ratio; CI: confidence Interval.

## 4 DISCUSSION

Many previous studies have described a relationship between metabolic syndrome and ADT.^7^, ^22^ Braga‐Basaria et al, reported that >50% of the men undergoing long‐term ADT predisposed them to higher cardiovascular risk.^7^ In addition, Marin et al, suggested that testosterone deficiency was significantly correlated with HTN, obesity, and hyperlipidemia.^23^ However, another study reported that associations between cerebral infarction and ADT are irrelevant―Alibhai et al, reported that ADT was associated with decreased risk for cerebral infarction (adjusted HR 0.88; *P* = 0.001).^15^ As mentioned in a recent meta‐analysis, a study reporting that ADT was not related to cerebral infarction included patients who underwent radical prostatectomy, whereas a significant relationship between ADT and stroke was observed after removing patients undergoing prostatectomy or radiotherapy.^24^

A few observational studies have shown that the risk for cerebral infarction increases in patients who undergo ADT.^7^, ^13^, ^14^ In a nested case‐control analysis using the United Kingdom's General Practice Research Database, Azoulay et al, reported that ADT users were at an increased risk for stroke/transient ischemic attack (TIA) (GnRH agonists; relative risk [RR] 1.18, oral antiandrogens; RR 1.47, bilateral orchiectomy; RR 1.77).^13^ In addition, Jespersen et al, reported that endocrine hormonal therapy was associated with an increased risk for cerebral infarction, but there was no association between orchiectomy and an increased risk for cerebral infarction.^12^ However, our results are not consistent with those of previous studies.

In this study, it is noteworthy that there was a difference in cerebral infarction incidence between ADT and non‐ADT patients in the crude cohort (annual incidence, 22.8 vs. 14.6 per 1000 person‐years; *P* < 0.001), which appears to be similar to that of previous studies. However, we found that there was serious selection bias between the two groups, which means significant differences in comorbidities between the ADT and the non‐ ADT groups. As previously reported, medical conditions, such as DM, HTN, PVD, and arterial fibrillation, are well‐known risk factors for cerebral infarction. Therefore, we assumed that if the comorbidities of both groups were similarly adjusted, the effect of ADT on cerebral infarction could be more accurately assessed. Our results demonstrated no increased risk for cerebral infarction in patients with PC treated with ADT compared with those who did not undergo ADT after adjusting for potential confounders including age, HTN, DM, CHF, PVD, atrial fibrillation, and medication status. This finding is consistent with the observation described by Chung et al, who reported that neither combined androgen blocker nor oral antiandrogen monotherapy was related to the risk for cerebral infarction among PC patients.^16^

In this study, we excluded patients who were treated with androgen receptor‐targeting agents or GnRH antagonist for the following reasons. The GnRH antagonists abiraterone acetate and enzalutamide were approved by the Korean Ministry of Food and Drug Safety in Korea in 2012 and 2013, respectively. Because reimbursement is only provided to patients when next‐generation androgen receptor‐targeting agents are used after chemotherapy, few patients qualified. In addition, the Korean health insurance system did not offer reimbursement to patients who were treated with GnRH antagonist until 2016. Therefore, in the data collection procedure, it is estimated that only 55 patients actually received next‐generation androgen receptor‐targeting agents and 230 received GnRH antagonists. It is controversial whether GNRH antagonists are more cardioprotective than GnRH agonists.^25^, ^26^, ^27^ If a larger sample size can be accumulated in Korea, a comparative study of GnRH antagonist versus GnRH agonist will be possible.

In our study, the incidence of cerebral infarction was calculated to be 14.6 cases per 1000 person‐years in the non‐ADT group. This result is comparable with a previously published study investigating the rate of cerebral infarction in men 65‐74 years of age, with a quoted incidence of 17.7 cases per 1000 person‐years in Korea.^28^ Meanwhile, the incidence of cerebral infarction was estimated to be 14.9 cases per 1000 person‐years in the ADT group. A notable finding of our study was that the duration of ADT was not associated with the risk for cerebral infarction in the Cox proportional hazard analysis. While only one previous study investigated the effect of ADT duration on the risk for cerebral infarction,^13^ an Asian study demonstrated that ADT was correlated with cerebral infarction, reporting that ADT duration was not associated with cerebral infarction.^14^

To the best of our knowledge, this study had the largest sample size investigating the association between ADT and cerebral infarction in Asians. Results of this investigation are meaningful considering that most studies examining this topic have addressed non‐Asian populations. Two previous studies have investigated the risk for cerebral infarction after ADT in an Asian population. First, during a 5‐year follow‐up in a prospective study involving 365 Chinese PC patients, Chung et al, reported that ADT was not correlated with the risk for cerebral infarction (adjusted HR 1.09 [95% CI 0.80‐1.50]).^16^ Second, Teoh et al, presented conflicting results in their population‐based study of Chinese living in Hong Kong (n = 452 patients).^14^ However, these Asian studies had the limitation of relatively small sample sizes. The present large‐population cohort study has several strengths. First, it was the first nationwide population‐based study to demonstrate that ADT is not associated with cerebral infarction in Asian patients with PC. Second, our findings suggest that underlying diseases, such as HTN, MI, RD, atrial fibrillation, MI, dementia, and PVD, may be risk factors for cerebral infarction in patients with PC. Third, our study included the unique descriptions of cohort data from the entire Korean PC population rather than data from selected or registered patients from trials, specific insurance claim providers, or sponsored registries. Therefore, results of this study reflect the real‐world clinical practice pattern of ADT use on a nationwide scale.

Despite these strengths, however, there were some limitations that should be addressed. First, our study had a retrospective design based on claims data, and our criteria for cerebral infarction relied on diagnostic codes. Second, this study did not include information regarding prognostic lifestyle factors including history of smoking or alcohol intake, or clinical data such as the Gleason score, prostate‐specific antigen levels, or tumor stage. Third, the possibility of code errors may exist in NHIS database. For example, although we excluded patients with a history of cerebral diseases, our definition of cerebral infarction may have included cerebral hemorrhagic events because the cerebral infarction subtype was not always specified in the claims data.^29^ Fourth, because the NHIS database used in this study yielded a maximum of 10 years of data, the follow‐up period was limited to 10 years. Nevertheless, previous studies have also reported a mean follow‐up period of between 3.3 and 3.9 years, and the longest reported follow‐up period was similar to that in our study.^12^, ^13^, ^30^ Finally, we did not analyze the risk for cerebral infarction according to ADT type.

In conclusion, our study found no significant difference in the risk for cerebral infarction between Korean patients with PC who did and did not undergo ADT, even after adjusting for comorbidities. Because ADT is not associated with an increased risk for subsequent stroke, this study provides PC patients with increased opportunities and access to ADT, as well as providing useful information for physicians weighing the benefits and risks of ADT. If an additional large sample‐size prospective study is performed, the hypotheses proposed here could be validated.

## DISCLOSURES

All authors have nothing to disclose.

## AUTHORS’ CONTRIBUTIONS

All those named as authors (BST, BJJ, HC, JHB, and JYP) have made a sufficient contribution to the work as follows: BST and JYP designed this study. BST, BJJ prepared the manuscript. BST and JYP reviewed and analyzed the data. BST carried out the statistical analysis. All authors performed critical review of this manuscript.

## Data Availability

I confirm that my article contains a Data Availability Statement even if no data is available (list of sample statements) unless my article type does not require one.
