# Supplementary material for: Is androgen deprivation therapy associated with cerebral infarction in patients with prostate cancer? A Korean nationwide population‐based propensity score matching study
Source: Cancer Med. 2019 Jun 10;8(9):4475–83. doi: 10.1002/cam4.2325 (PMC6675721; doi:10.1002/cam4.2325)
Supplement: Supplementary file 1 [file CAM4-8-4475-s001.docx]

**S1 Table. Definition of treatment code**

| Medication |  | Billing Code |
| --- | --- | --- |
| **ADT** | | |
| GnRH agonists | Leuprolide | 182601BIJ, 182602BIJ, 182604BIJ, 182610BIJ, 182630BIJ |
|  | Goserelin | 167202BIJ, 167201BIJ, |
|  | Triptorelin | 244901BIJ, 244902BIJ, 244930BIJ, 467501BIJ |
| Antiandrogens | Bicalutamide | 117201ATB, 117202ATB |
|  | Cyproterone | 139401ATB, 398400ATB |
|  | Flutamide, | 162101ATB |
| GnRH antagonists | Degarelix | 624401BIJ, 624402BIJ |
| Estrogens | Estramustine | 155101ACH |
| Bilateral orchiectomy |  | R3851, R3852, |
| **Other Medication** | | |
| Antiplatelet |  | 111001ATE, 110702ATB, 110701ATB, 111001ACE, 111003ACE, 136901ATB, 495201ATB, 501501ATB, 133202ATB, 133201ATB, 244101ACE, 597302ATB, 597302ATB, 615901ATB |
| Anticoagulant |  | 249103ATB, 249105ATB, 511401ATB, 511402ATB, 511403ATB, 511404ATB, 613701ACH, 613702ACH, 617001ATB, 617002ATB, 643601ATB, 643602ATB, 643603ATB, 450101BIJ |
| Statin |  | 216601ATB, 216603ATB, 216604ATB, 111501ATB, 111502ATB, 454001ATB, 454002ATB, 454003ATB, 470901ATB, 227801ATB, 227802ATB, 227803ATB, 230202BIJ, 185801ATB, 472300ATB, 472400ATB, 162403ATR, 162401ACH, 162402ACH |
| SSRI |  | 209306ATR, 209305ATR, 209304ATR, 209302ATB, 209301ATB, 161502ACH, 161501ACH, 161502ATD, 161504ACR, 161502ATD |
| Antipsychiatric |  | 420003ATB, 420004ATB, 420002ATB, 451501ATB, 451503ATB, 451502ATB, 451501ATD, 451502ATD, 451503ATB, 451504ATB, 451505ATB, 137502ATB, 137501ATB, 137501ATB, 137502ATB, 204001ATD, 204002ATD, 204001ATB, 204002ATB, 204004ATB, 503201ATR, 503202ATR, 503203ATR, 378602ATB, 378603ATB, 378601ATB, 378604ATB, 378605ATR, 378606ATR, 378607ATR, 378608ATR, 378609ATR,  224201ATB, 224202ATB, 224203ATB, 224204ATB |

gonadotropin-releasing hormone, GnRH; Androgen deprivation therapy, ADT

**S2 Table. Definition of diagnosis code**

| Comorbidities | ICD-10-CM code and definition |
| --- | --- |
| Cerebrovascualr disease | G45.x, G46.x, H34.0, I60-I69 |
| Hypertension^a^ | I10-I13, I15; and minimum 1 prescription of anti-hypertensive drug (thiazide, loop diuretics, aldosterone antagonist, alpha-/beta-blocker, calcium-channel blocker, angiotensin-converting enzyme inhibitor, angiotensin II receptor blocker). |
| Diabetes mellitus^a^ | E10-E14; and minimum 1 prescription of anti-diabetic drugs (sulfonylureas, metformin, meglitinides, thiazolidinediones, dipeptidyl peptidase-4 inhibitors, α-glucosidase inhibitors and insulin). |
| Congestive heart failure | I09.9, I11.0, I13.2, I25.5, I42.0, I42.5-I42.9, I43.x, I50.x, P29.0 |
| Transient ischemic attack | G458, G459 |
| Peripheral vascular disease | I70-I71, I73.x, I77.1, I79.0, I79.2, K55.1, K55.8, K55.9, Z95.8, Z95.9 |
| Myocardial infarction | I21, I22, I25.2 |
| Dementia (Alzheimer's Disease) | F00.x-F03.x, F05.1, G30.x, G31.x |
| Atrialfibrillation and flutter | I 48 |

All variables except hypertension and diabetes mellitus were defined when patients had one or more diagnoses during hospitalization or at outpatient clinic.

^a^ Hypertension and diabetes mellitus were identified when patients had ≥1 diagnoses during hospitalization or ≥2 diagnoses at outpatient clinic for preventing overestimation of diagnosis.
